# Targeting gut-microbiota for gastric cancer treatment: a systematic review

**DOI:** 10.3389/fmed.2024.1412709

**Published:** 2024-08-02

**Authors:** Amir Marashi, Saina Hasany, Sadra Moghimi, Reza Kiani, Sina Mehran Asl, Yasamin Alavi Dareghlou, Parsa Lorestani, Shirin Varmazyar, Fatemeh Jafari, Shakiba Ataeian, Kiana Naghavi, Seyed Mohammad Sajjadi, Negar Haratian, Arman Alinezhad, Aida Azhdarimoghaddam, Seyed Kiarash Sadat Rafiei, Mahsa Asadi Anar

**Affiliations:** ^1^School of Medicine, Tehran University of Medical Sciences, Tehran, Iran; ^2^Student Research Committee, Islamic Azad University Tehran Medical Sciences, Tehran, Iran; ^3^Student Research Committee, Islamic Azad University Tehran Medical Sciences, Mashhad, Iran; ^4^School of Medicine, Shahroud Azad University of Medical Sciences, Shahroud, Iran; ^5^Student Research Committee, School of Medicine, Alborz University of Medical Sciences, Alborz, Iran; ^6^School of Medicine, Mazandaran University of Medical Sciences, Sari, Iran; ^7^School of Pharmacy, Mazandaran University of Medical Sciences, Sari, Iran; ^8^Student Research Committee, Shahid Beheshti University of Medical Sciences, Tehran, Iran; ^9^Student Research Committee, Mashhad University of Medical Sciences, Mashhad, Iran; ^10^School of Medicine, Shahid Beheshti University of Medical Sciences, Tehran, Iran; ^11^Student Research Committee, School of Medicine, Shahid Beheshti University of Medical Sciences, Tehran, Iran

**Keywords:** gastric cancer, gut microbiota, immunotherapy, immune regulation, microbial metabolites

## Abstract

**Background:**

Preclinical research has identified the mechanisms via which bacteria influence cancer treatment outcomes. Clinical studies have demonstrated the potential to modify the microbiome in cancer treatment. Herein, we systematically analyze how gut microorganisms interact with chemotherapy and immune checkpoint inhibitors, specifically focusing on how gut bacteria affect the pharmacokinetics and pharmacodynamics of cancer treatment.

**Method:**

This study searched Web of Science, Scopus, and PubMed until August 2023. Studies were screened by their title and abstract using the Rayyan intelligent tool for systematic reviews. Quality assessment of studies was done using the JBI critical appraisal tool.

**Result:**

Alterations in the gut microbiome are associated with gastric cancer and precancerous lesions. These alterations include reduced microbial alpha diversity, increased bacterial overgrowth, and decreased richness and evenness of gastric bacteria. *Helicobacter pylori* infection is associated with reduced richness and evenness of gastric bacteria, while eradication only partially restores microbial diversity. The gut microbiome also affects the response to cancer treatments, with higher abundances of *Lactobacillus* associated with better response to anti-PD-1/PD-L1 immunotherapy and more prolonged progression-free survival. Antibiotic-induced gut microbiota dysbiosis can reduce the anti-tumor efficacy of 5-Fluorouracil treatment, while probiotics did not significantly enhance it. A probiotic combination containing *Bifidobacterium infantis, Lactobacillus acidophilus, Enterococcus faecalis*, and *Bacillus cereus* can reduce inflammation, enhance immunity, and restore a healthier gut microbial balance in gastric cancer patients after partial gastrectomy.

**Conclusion:**

Probiotics and targeted interventions to modulate the gut microbiome have shown promising results in cancer prevention and treatment efficacy.

**Systematic review registration:** https://osf.io/6vcjp.

## Introduction

Gastric cancer (GC) is among the most critical health issues worldwide. Beyond improvements in diagnosis, surveillance, and treatment over the past years, it is ranked as the fifth cancer in incidence and fourth in cancer mortality cause. The last report from 2020 shows the incidence of over 1 million new cases per year and more than 768/000 deaths annually. However, the incidence varies worldwide, with the highest cases occurring in Asia, with more than 820/000 new cases and 576/000 mortality (1). It is estimated that the statistics will rise with all efforts, and in recent years, the incidence of young adults has been increasing. The effect of *Helicobacter pylori (Hp)* is known already. Moreover, other risk factors such as genetics, tobacco use, alcohol consumption, and other lifestyle elements also play an essential role in the pathogenesis of the disease in both high-income and low-income countries. This may explain the possible rise in the disease incidence in the future (2, 3).

Patients at early stages are mostly asymptomatic or may have non-specific symptoms such as dyspepsia. At advanced stages, the tumor might demonstrate abdominal pain, weight loss, anorexia, or might exhibit complications of the tumor, such as hematemesis for ulcerated tumors or persistent vomiting in gastric outlet obstruction (4). This is why, in many countries, patients may present with advanced disease without efficient screening systems for GC, making the treatment more complicated.

More than 90% of the tumors arise sporadically, and the remnants have hereditary origins (5). According to Lauren’s classification of tumors, which is still a widely known histopathological classification besides the World Health Organization (WHO), there are three subtypes of tumor, including Intestinal, Diffuse (signet ring), and indeterminate (6). WHO also divides GC tumors into groups based on their origin: Adenocarcinomas, squamous cell carcinoma, non-Hodgkin lymphoma, Gastrointestinal stromal tumor (GIST), and Neuroendocrine tumors, which tend to occur less frequently. Adenocarcinomas are also poorly cohesive, Mucinous, Papillary, and tubular. Intestinal and Tubular subgroups are the most common types among the patients (7).

As we mentioned above, due to the late diagnosis in advanced stages, GC is mostly recognized at advanced stages with metastasis, decreasing the survival rate and complicating the treatment plan. Over the past decades, many therapeutic approaches have been introduced based on surgical, medical, and a combination. Adjuvant or neo-adjuvant therapies, along with radical surgery, improve the survival of resectable tumors (8). Despite the progress, the challenge of unresectable advanced-stage or progressive metastatic tumors remains a primary concern (9, 10). In recent years, targeted therapies based on mononuclear antibodies have also been applied in this field (11, 12). Beyond all of these efforts, because of the complexity of the disease and heterogeneity of tumors related to the genetics of patients and environmental factors, the overall survival is still not hopeful and hardly achieves one year (13). In an attempt to find the reasons for failure in chemotherapy regimens and targeted therapies, several studies have demonstrated that the arrangement of the gut microbiome can impact the host’s reaction to therapy (14, 15).

The role of gut microbiota, the collection of colonized microorganisms in the gastrointestinal lumen, on a healthy immune system has been studied in past years. It has been shown to have an essential effect on human health and regulates various aspects of it, such as host immunological responses, energy metabolism, elimination of pathogens, and oncogenesis (16). The dysbiosis of this system may affect several diseases, including GC (17, 18). Previous research has proven that infection with *helicobacter pylori*, a known and significant risk factor of the condition, can cause alterations in the microbiome. These changes have been found to contribute not only to gastric atrophy but also to an increased likelihood of developing gastric cancer, as observed in both animal and human models (19, 20). According to the study conducted by Miao et al. (21), it has been observed that the population of organisms changes at the various stages of the disease, thus presenting potential opportunities for focused treatment interventions. Although the impact of gut microbiota on chemotherapy and targeted immune therapy has been investigated, its precise mechanisms and effects remain uncertain.

Hence, this study aims to evaluate the impact of explicitly targeting the gut microbiota on the treatment results of gastric cancer through a systematic review and meta-analysis conducted for the first time.

## Methods

This systematic review follows the principles of the Preferred Reporting Items for Systematic Reviews and Meta-Analyses (PRISMA2020) statement (22). The study protocol has been registered on the Open Science Framework (OSF) (registration doi: 10.17605/OSF.IO/6VCJP).

### Search strategy

We collected original articles in this field by searching through PubMed, Google Scholar, Web of Science, and Scopus databases for English language literature published up to the 12th of August, 2023. The search was conducted based on “Gastric or stomach cancer,” “Gastric or stomach neoplasm,” AND “Gut microbiota” or “Microbiome” as keywords. The search strategy is mentioned in Table 1. Furthermore, duplicated records were omitted using EndNote ver.21. To identify other suitable studies, we also reviewed the references of relevant papers and reviews on the topic (see Table 2).

**Table 1:** Curated search strategies for chosen databases.

Data base	Search strategy	Results
Pubmed	(“Gastrointestinal Microbiome”[MeSH Terms] OR “Gut Microbiome”[Title/Abstract] OR “Gut Microbiomes”[Title/Abstract] OR “Gut Microflora”[Title/Abstract] OR “Gut Microbiota”[Title/Abstract] OR “Gut Microbiotas”[Title/Abstract] OR “Gut Flora”[Title/Abstract]) AND (“Stomach Neoplasms”[MeSH Terms] OR “Stomach Neoplasms”[Title/Abstract] OR “Stomach Neoplasm”[Title/Abstract] OR “Gastric Neoplasms”[Title/Abstract] OR “Gastric Neoplasm”[Title/Abstract] OR “Stomach Cancers”[Title/Abstract] OR “Gastric Cancer”[Title/Abstract] OR “Gastric Cancers”[Title/Abstract] OR “Stomach Cancer”[Title/Abstract])	259
WOS	#1 TS = (“Gut Microbiotas”) OR TS = (“Gastrointestinal Microbiome”) OR TS = (“Gut Microbiome”) OR TS = (“Gut Microflora”) Results 3,632 2# TS = (“Gastric Neoplasm”) OR TS = (“Gastric Cancer”) OR TS = (“Stomach cancer”) OR TS = (“Stomach Neoplasms”) Results 102,679	21
Scopus	(TITLE-ABS-KEY (“Gut Microbiotas”) OR TITLE-ABS-KEY (“Gastrointestinal Microbiome”) OR TITLE-ABS-KEY (“Gut Microbiome”) OR TITLE-ABS-KEY (“Gut Microflora”)) AND (TITLE-ABS-KEY (“Stomach Neoplasms”) OR TITLE-ABS-KEY (“Gastric Neoplasm”) OR TITLE-ABS-KEY (“Gastric Cancers”) OR TITLE-ABS-KEY (“Stomach cancers”) OR TITLE-ABS-KEY (“Gut Flora”) OR TITLE-ABS-KEY (“Stomach Neoplasms”))	845
Total		1,125

**Table 2:** Summary findings of studies included in the systematic synthesis.

Author	Year	Country	Population	Sex	Type of Study	Bacteria species	Organ	Gastric cancer description	Outcome	Conclusion	Quality Of evidence
Zihan et al. (23)	2023	China	117 patients	Male:90 Female:27	Prospective cohort study	*Lactobacillus, Bacillota, Bacteroidota* and *Actinobacteria, Proteobacteria* outnumbered *Actinobacteria*	Gastric	Metastatic/unresectable HER2-negative gastric/gastroesophageal junction adenocarcinoma	The nature of the gut microbiota influences the efficacy of various therapies in patients with HER2-negative advanced gastric cancer. Increased levels of Lactobacillus are linked to improved outcomes with anti-PD-1/PD-L1 immunotherapy and extended progression-free survival (PFS). Patients with increased levels of Lactobacillus exhibited more variety in their gut microbiota and showed an increase in metabolic pathways associated with amino acid and energy metabolism. In the group receiving immunotherapy in addition to chemotherapy, a decreased presence of Streptococcus was linked to improved response and extended progression-free survival (PFS). The gut microbiota profiles associated with treatment response differ between chemotherapy, immunotherapy, and combination therapy groups, indicating an intricate interaction. *Lactobacillus* has the potential to enhance the effectiveness of immunotherapy in treating gastric cancer.	The study highlights the importance of gut microbiota in cancer treatment, with Lactobacillus potentially playing a crucial role. Focusing on these bacteria could potentially improve treatment outcomes and enhance patient outcomes.	High
Jones et al. (24)	2017	United States	It was tested on *Drosophila melanogaster*	-	Experimental study	*Helicobacter pylori*	Gastric	-	Expression of the *H. pylori* virulence factor In the Drosophila gut model, CagA stimulates abnormal growth of epithelial cells, a characteristic of gastric cancer in people with *H. pylori* infection. CagA expression results in dysbiosis of the gut microbial population. The altered microbiota caused by CagA expression leads to increased cell proliferation, indicating that dysbiosis may worsen stomach cancer development in *H. pylori* infection.	The gut microbiome composition influences treatment response in HER2-negative advanced gastric cancer patients. High *Lactobacillus* abundance leads to better response and longer progression-free survival. Lower Streptococcus abundance in immunotherapy plus chemotherapy group improves response and PFS. *Lactobacillus* may be a potential adjuvant agent for immunotherapy efficacy.	High
Miao et al. (21)	2022	China	51 patients	Female:51	Cross-sectional study		Gastric	*Enterococcus Fusicatenibacter Faecalibacterium, RoseburiaLachnoclos Lachnoclostridium, Tyzzerella_3, Roseburia, Butyricicoccus, and Dorea*	Reduced gut microbial alpha diversity (richness and diversity) and altered dissimilarity of the microbial community structure were found in the gastric mucosal atypical hyperplasia and gastric cancer groups compared to the superficial gastritis group. Several bacterial genera were enriched in different stages: (a) Superficial gastritis group: *Dorea*,*Erysipelotrichaceae*_unclassified, *Ruminococcaceae*_unclassified, *Fusicatenibacter*, *Faecalibacterium, Roseburia, Lachnoclostridium*, *Butyricicoccus* (b) Atrophic gastritis group: *Tyzzerella_3*, *Actinomyces*, *Lachnospiraceae_unclassified* (c) Gastric mucosal atypical hyperplasia group: *Burkholderiales_unclassified, Peptoniphilus, Alloprevotella, Prevotella_7* (d) Gastric cancer group: *Porphyromonas, Scardovia, Halomonas, Actinobacteria_unclassified, Bergeyella, Enterococcus* The genera *Scardovia* and *Halomonas* were newly associated with gastric cancer. The metabolic pathways of Genetic Information Processing and Circulatory System were more abundant in the gastric cancer group compared to non-cancer groups.	The study explores the connection between gut microbiota and cancer treatment response. It found that certain microbial taxa, like *L. mucosae* and *L. salivarius*, are associated with better immunotherapy response. Lactobacillus abundance in responders is linked to immunotherapy effects, while higher alpha-diversity indicates a more diverse gut microenvironment. The combination of immunotherapy and chemotherapy has a distinct microbiome signature. Streptococcus abundance in responders correlates with shorter progression-free survival. These findings suggest gut microbiota could be a potential biomarker for cancer diagnosis and patient stratification.	Moderate
Oh et al. (25)	2015	South Korea	32		Randomized controlled trial	*H. pylori, Streptococcus faecium Bacillus subtilis* *phyla, Bacillota, Bacteroidota, and Proteobacteria*	Gastric	Chronic gastritis, gastric and duodenal ulcers as well as gastric cancer	probiotic supplementation during *H. pylori* eradication therapy helped reduce the disruption of gut microbiota caused by the antibiotics. Maintaining a healthy, balanced gut microbiota is important, as dysbiosis (imbalance) of gut microbiota has been linked to several diseases, including gastric cancer. While the study did not directly evaluate gastric cancer risk, maintaining gut microbiota balance by using probiotics during *H. pylori* eradication may help reduce risk factors associated with gastric cancer development.	Probiotic supplementation can reduce the antibiotic-induced alteration and imbalance of the gut microbiota composition. This effect may restrict the growth of antibiotic-resistant bacteria in the gut and improve the *H. pylori* eradication success rate.	Moderate
Park et al. (26)	2018	South Korea	138 patients	Male:63	Cross-sectional study	*H. pylori* *Rhizobiales* *Cyanobacteria*	Gastric	*H. pylori*-negative CSG, *H. pylori*-negative IM, *H. pylori*-negative cancer, *H. pylori*-positive CSG, *H. pylori*-positive IM, and *H. pylori*-positive cancer	The relative abundance of the bacterial taxa *Rhizobiales* was higher in patients with *H. pylori*-negative intestinal metaplasia compared to those with *H. pylori*-negative chronic superficial gastritis or *H. pylori*-negative gastric cancer. Genes encoding type IV secretion system (T4SS) proteins, which are essential for transferring the CagA virulence factor from *H. pylori* into human gastric epithelial cells, were highly prevalent in the metagenome of patients with intestinal metaplasia. The authors hypothesized that the abundance of T4SS genes in intestinal metaplasia, mainly contributed by bacteria like *Rhizobiales* and *Neisseriaceae*, may facilitate horizontal gene transfer of T4SS genes to *H. pylori*, promoting gastric carcinogenesis through enhanced CagA translocation. The gastric microbiome composition after successful *H. pylori* eradication therapy resembled that of the *H. pylori*-negative intestinal metaplasia group, which was regarded as a high-risk group for gastric cancer development.	A study found that patients with *H. pylori*-negative intestinal metaplasia (IM) had a higher abundance of *Rhizobiales* bacteria compared to those with chronic superficial gastritis (CSG) or cancer. The metagenome of IM patients also showed high T4SS protein genes, potentially promoting gastric carcinogenesis. The study suggests that horizontal gene transfer between *H. pylori* and other bacteria may contribute to gastric cancer development.	High
Park et al. (27)	2019	South Korea	83	Male:39 Female:44	Cross-sectional study	*H. pylori* *Acidobacteriaceae, Burkholderiaceae, Neisseriaceae, Pasteurellaceae, Veillonellaceae, Bartonellaceae, Brucellaceae, unclassified Rhizobiales, Pseudomonadaceae, Sphingomonadaceae, Staphylococcaceae, and Xanthomonadaceae*		Patients with gastric neoplasms including carcinoma, mucosa-associated lymphoid tissue lymphoma, or adenoma; and (c) patients who underwent gastrectomy	Through weighted correlation network analysis identified two microbial modules (pink and brown modules) that were positively correlated with an advanced stage of gastric carcinogenesis (intestinal metaplasia with or without *H. pylori* infection). The pink and brown modules included various bacteria such as nitrosating/nitrate-reducing bacteria (e.g., *Neisseriaceae, Pasteurellaceae, Veillonellaceae, Pseudomonadaceae, Staphylococcaceae*), type IV secretion system (T4SS) protein gene-contributing bacteria (e.g., *Acidobacteriaceae, Burkholderiaceae, Neisseriaceae, Bartonellaceae, Brucellaceae, Rhizobiales, Pseudomonadaceae, Sphingomonadaceae, Xanthomonadaceae*), and other bacterial families like *Gordoniaceae, Tsukamurellaceae, Prevotellaceae, Cellulomonadaceae, Methylococcaceae*, and *Procabacteriaceae*. The abundance of bacterial taxa in the pink and brown modules was higher in patients with intestinal metaplasia (precancerous lesion) compared to those without intestinal metaplasia. In contrast, the blue module, which included *H. pylori*, was negatively correlated with intestinal metaplasia. The findings suggest that diverse intragastric bacteria, beyond just *H. pylori*, are associated with an advanced stage of gastric carcinogenesis, and these bacteria can be clustered into specific microbial network modules through weighted correlation analysis.	The study identifies two bacterial modules associated with gastric carcinogenesis, including *H. pylori* and other taxa. It also reveals that the abundance of these taxa decreases in patients with intestinal metaplasia. This contributes to a better understanding of the gastric microbiome and its association with gastric carcinogenesis.	High
Zheng et al. (29)	2019	China	100	Male:84 Female:16	Randomized controlled trial	*Bifidobacterium infantis, Lactobacillus acidophilus, Enterococcus faecalis and Bacillus cereus* *Streptococcus, Peptostreptococcus and Prevotella,* *Bifidobacterium* *Bacteroides, Faecalibacterium* and *Akkermansia*	Gastric	Gastric cancer	The probiotic combination containing *Bifidobacterium infantis, Lactobacillus acidophilus, Enterococcus faecalis* and *Bacillus cereus* significantly reduced inflammation indexes (leukocytes) and enhanced immunity indexes (lymphocytes) and nutrition indexes (albumin and total protein) in gastric cancer patients after partial gastrectomy compared to the control group. Gastric cancer had a strong influence on the microbial diversity in the stomach, enhancing the abundance of pathogens like *Streptococcus, Peptostreptococcus* and *Prevotella*, while reducing the probiotic *Bifidobacterium*. The probiotic combination reduced the *Bacillota /Bacteroidota* ratio in the gut microbiota of gastric cancer patients after gastrectomy compared to the control group, which is associated with a healthier state. At the genus level, the probiotic combination enhanced beneficial bacteria like *Bacteroides, Faecalibacterium* and *Akkermansia*, while lowering the pathogen Streptococcus in the gut of gastric cancer patients after surgery. Overall, the probiotic combination enhanced immune response, reduced inflammation severity, and helped restore a healthier gut microbial balance in gastric cancer patients after partial gastrectomy surgery.	Gastrectomy, a common treatment for gastric cancer, can cause severe physiological and microbial disorders. A study suggests using a probiotic combination can reduce side effects, improve health, and restore a healthier balance in the stomach and intestinal microbiota. Further research is needed to understand the underlying mechanisms.	High
Yuan et al. (30)	2018	China	32 mice	Female:32	Experimental study	*Escherichia shigella Enterobacter* *Bacillota Lachnospiracea_NK4 A136, Bacteroides, Odoribacter, Mucispirillum,* and *Blautia*		Colorectal cancer	Antibiotics treatment that disrupted the gut microbiota reduced the anti-tumor efficacy of 5-FU in the mice with colorectal cancer. Administration of probiotics along with 5-FU did not significantly increase the anti-tumor efficacy compared to 5-FU alone, although it improved body weight in mice at day 33. 5-FU treatment altered the diversity and composition of the gut microbiota, with increased abundance of certain bacterial genera like *Lachnospiraceae, Enterobacter, Escherichia-Shigella, Bacteroides*, etc. Functional analysis showed genes involved in amino acid metabolism, replication/repair, translation, and nucleotide metabolism were expressed lower in the antibiotics +5-FU group compared to other groups. The results suggest that gut microbiota dysbiosis induced by antibiotics may contribute to reduced anti-tumor efficacy of 5-FU, highlighting the potential role of gut microbiota in modulating chemotherapeutic drug responses in colorectal cancer.	The study reveals that gut microbiota imbalance can reduce the effectiveness of 5-Fluorouracil (5-FU) treatment in reducing tumor growth in colorectal cancer. The imbalance alters the composition and function of the microbiota, leading to increased harmful bacteria and decreased beneficial ones. Probiotics do not significantly alter the gut microbiota diversity, but change the types of bacteria present.	High
Yu et al. (31)	2023	China			Experimental study	*H. pylori*			The nanogenerators (Fe-HMME@DHA@MPN) can generate ROS like singlet oxygen and hydroxyl radicals under ultrasonication and acidic conditions mimicking the *H. pylori* infection microenvironment in the stomach. The ROS produced by these nanogenerators were effective in killing multidrug-resistant *H. pylori* strains and removing *H. pylori* biofilms *in vitro*. In a mouse model of *H. pylori* infection, treatment with these nanogenerators showed high therapeutic efficacy in eliminating the *H. pylori* infection without disrupting the normal gut microbiota.	A gastric acid-responsive ROS nanogenerator made from biocompatible DHA, tannic acid, HMME, and Fe (II, III) has been used to treat *H. pylori* infection in mice through sonodynamic and chemodynamic processes. The nanogenerator catalyzes H2O2 and produces ROS in an acidic environment, effectively eliminating drug-susceptible and drug-resistant *H. pylori* and biofilm without harming bacteria or cells.	Moderate
Watanabe et al. (32)	2020	Japan	29 *H. pylori*-infected patients	Male:24 Female:5	Case control study	*H. pylori*	Gastric Cancer	Early GC	*H. pylori* infection was associated with reduced richness and evenness of gastric bacteria compared to *H. pylori*-negative patients. Several genera like *Blautia, Ralstonia, Faecalibacterium, Methylobacterium*, and *Megamonas* were depleted in *H. pylori*-positive patients. *H. pylori* eradication only partially restored microbial diversity, and those 5 genera remained depleted compared to *H. pylori*-negative patients. The gastric microbiota composition clustered into three distinct groups based on *H. pylori* status: negative, pre-eradication, and post-eradication.	*Helicobacter pylori*, a major cause of gastric cancer, affects over half of the global population. Drug resistance is affecting antibiotic-based triple therapy efficacy. Other gastric microbiomes also contribute to GC tumorigenesis. Early GC patients risk metachronous GC, and dysbiosis may persist post-eradication.	High
Wang et al. (33)	2016	China	315	Male:190	Case–control study	*H. pylori*	Gastric	Chronic gastritis Gastric Cancer	There was an increased bacterial load (overgrowth) in the gastric mucosa of patients with gastric cancer compared to chronic gastritis. The structure of the microbial communities was more diversified in gastric cancer patients. Five bacterial genera (*Lactobacillus, Escherichia-Shigella, Nitrospirae*, *Burkholderia fungorum,* and *Lachnospiraceae* uncultured) were enriched in gastric cancer patients. The presence of *Helicobacter pylori* infection was associated with increased bacterial load but did not significantly alter the relative abundance of other bacteria. The altered microbiota in gastric cancer, with increased bacterial quantity, diversified communities, and enrichment of bacteria with potential cancer-promoting activities, could contribute to gastric carcinogenesis.	The study aimed to characterize gastric microbiota in cancer, finding an increased number of diverse bacteria. The altered microbiota may have cancer-promoting activities, but mechanisms and pathways remain unclear.	Moderate
Wang et al. (34)	2019	China	313	Male:165 Female:148	Cohort Study	*H. pylori*		Gastric ulcer, gastric cancer, and many nongastrointestinal disorders	alterations in the gut microbiome composition and functions in *H. pylori* positive individuals compared to *H. pylori* negative individuals. Specific microbial species like *Prevotella copri,* linked to inflammatory conditions like rheumatoid arthritis, were enriched in *H. pylori* positive individuals. Microbial genes/pathways related to vitamin B12 biosynthesis were diminished in *H. pylori* positive individuals, who also had lower blood vitamin B12 levels. Overall, the study suggests *H. pylori* infection leads to dysbiosis of the gut microbiome, which may contribute to the downstream effects and disease risks associated with *H. pylori*, including gastric cancer, though this was not directly studied.	The study shows that *Helicobacter pylori* infection impacts the microbial makeup and function of the human intestines in the Chinese population. The study also shows variations in the prevalence of immunologically associated bacteria *P. copri.* Dysbiosis of gut microbiota connected to HPI can elevate the likelihood of VB12 insufficiency, offering fresh perspectives on the relationship between *H. pylori* and the microecology of the host’s gastrointestinal system.	High
Turati et al. (35)	2023		1,600		Case report????? (case control)		stomach	There were 946 cases of oral cavity/pharynx cancer, 198 cases of nasopharynx cancer, 304 cases of esophageal cancer, and 230 cases of stomach cancer. Over 4,000 individuals treated to the same hospitals for acute nonneoplastic and non-diet-related diseases were chosen as control subjects.	No association was observed between intake of most prebiotic fibers (inulin-type fructans, fructooligosaccharides like kestose, nystose, 1F-β-fructofuranosylnystose, and galactooligosaccharide stachyose) and risk of stomach cancer. high intake of the galactooligosaccharide raffinose was associated with a reduced risk of stomach cancer. Specifically, the odds ratio for stomach cancer in the highest vs. lowest tertile of raffinose intake was 0.6 (95% CI: 0.3–0.9).	Fiber intake may lower digestive tract cancer risk by modifying gut microbiota. However, no data exists on specific fiber fractions with prebiotic activity. No association was found between prebiotic intake and oral cavity, pharynx, nasopharynx, and esophagus cancers.	High
Song et al. (36)	2017	China	35	Male:35	Experimental study	*Desulfovibrio, Mucispirillum, Odoribacter, Lactobacillus*			Pretreatment with the probiotic cocktail *Bifico* ameliorated colitis and reduced tumor formation in the CAC mouse model. *Bifico* treatment alleviated weight loss, reduced tumor multiplicity and size, and lowered expression of pro-inflammatory genes like Tnfa, Il1b, Il6, and Ptgs1. *Bifico* altered the composition of the gut microbiota, decreasing abundance of genera like *Desulfovibrio, Mucispirillum, Odoribacter* and increasing *Lactobacillus*. The changes in gut microbiota induced by *Bifico* correlated with reduced expression of CXCR2 ligand chemokines like CXCL1, CXCL2, CXCL3, CXCL5 which promote tumor progression.	Individuals with inflammatory bowel disease are at high risk of developing colitis-associated cancer (CAC). Probiotic mixture *Bifico* has shown efficacy in chemopreventive effects on CAC. However, causal relationship between changes in transcriptome and microbial community and attenuated tumorigenesis is unclear. Further studies are needed.	Moderate
Qi et al. (37)	2022	China	124 patients		Cohort study		Colorectal cancer	Colorectal cancer	There were no significant differences in gut bacterial alpha diversity between moderately and poorly differentiated CRC groups. At the genus level, 9 bacterial genera were more abundant in the poorly differentiated CRC group, including *Bifidobacterium, Oscillospiraceae*, and *Eisenbergiella.* 6 bacterial genera were more abundant in the moderately differentiated CRC group, including *Megamonas, Erysipelotrichaceae_UCG-003,* and *Actinomyces.* A random forest model using differential gut bacteria could predict poorly differentiated CRC with 100% accuracy, with *Pseudoramibacter, Megamonas* and *Bifidobacterium* being the most important bacterial predictors. The study suggests that gut bacterial composition is related to the degree of pathological differentiation in CRC, and specific bacteria may serve as biomarkers for predicting poorly differentiated CRC.	Colorectal cancer mortality is high, malignant malignancy is high, and prognosis poor. Gut flora affects pathological differentiation, with different bacterial flora used as biomarkers for poorly differentiated colorectal cancer.	High

### Inclusion criteria

After excluding the animal studies, the remaining studies were included for the review if the study follows the PICOS:

P: population: gastric cancer patients.

I: intervention: using gut microbiota in treatment regimen.

C: the control group: gastric cancer patients who receive treatments regardless of gut microbiota.

O: outcome: patient’s progression-free survival and responsiveness to treatment.

S: study design: English language RCTs

### Study selection and quality assessment

Two reviewers (SH, SM), Using the RAYYAN intelligent tool for systematic reviews, analyzed and screened titles and abstracts to identify similar papers in a blinded manner. Full texts of them were obtained to assess the qualification of the “Yes” and “Maybe” groups. In case of conflicts, a third reviewer was involved and then reached an agreement to overcome differences and disagreements. Conflicts have been resolved through discussion between them. For each included study, the quality assessment and risk of bias were performed using JBI’s critical appraisal tools.^1^

## Result

### Study characteristics

**Figure 1:**
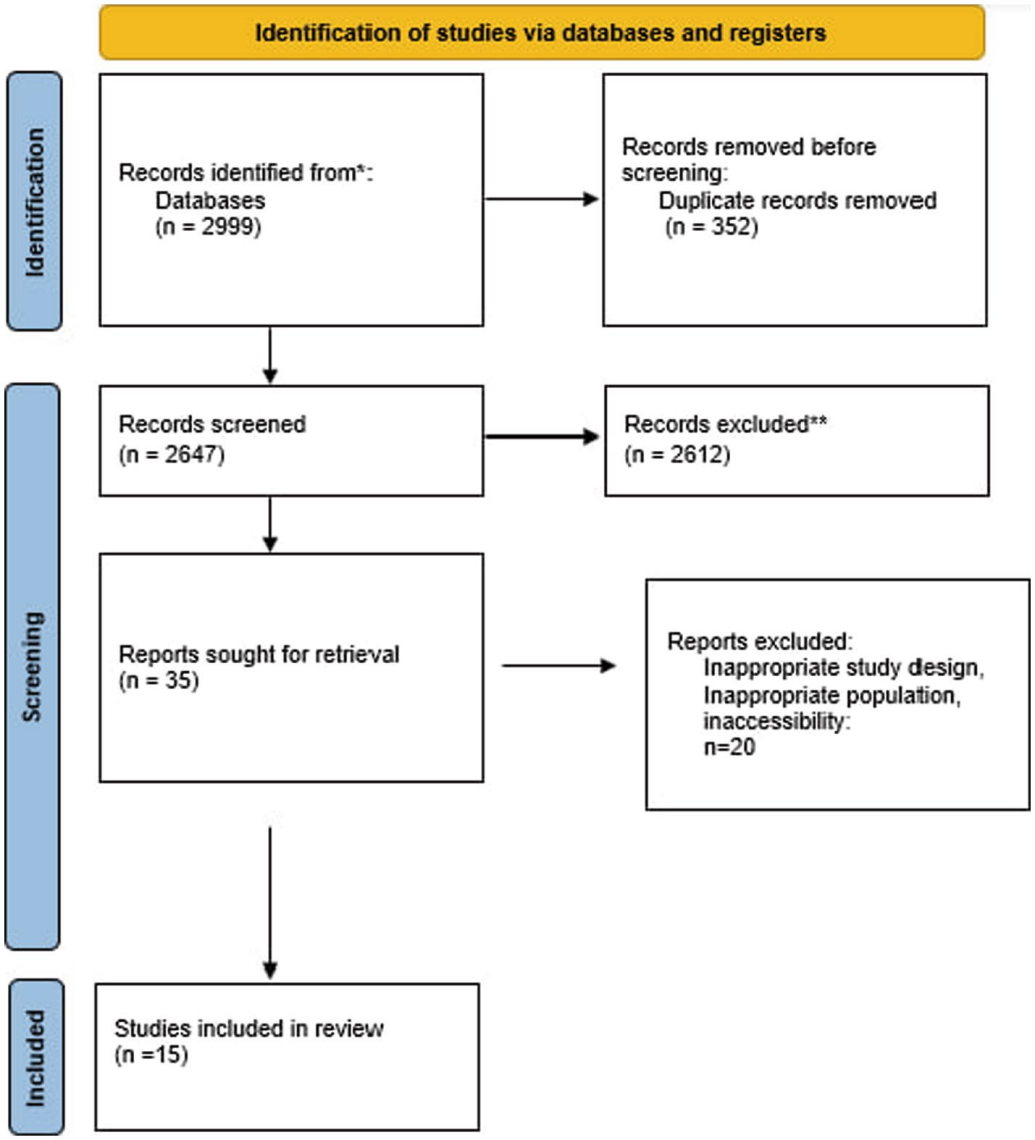
Flow diagram of the study selection procedure. The PRISMA approach to meta-analysis’s flow diagram template was used.

Our search strategies yielded 2,999 studies across databases (Figure 1). After removing duplicates and excluding articles that did not meet our criteria, 15 studies were included in this systematic review, comprising eight observational studies (4 cross-sectional studies, two case–control studies, and 2 cohort studies), five experimental studies, and two randomized controlled trials. The studies were conducted in various countries, including China (7 studies), South Korea (3 studies), the United States (1 study), Japan (1 study), and multinational collaborations (2 studies). Sample sizes ranged from 19 to 1,600 participants, with some studies involving animal models or *in vitro* experiments.

### Gut microbiome and gastric cancer

Several studies reported alterations in the gut microbiome composition and diversity associated with gastric cancer or precancerous lesions. Miao et al. (21) found reduced microbial alpha diversity and altered community structure in patients with gastric mucosal atypical hyperplasia and gastric cancer compared to those with superficial gastritis. Specific bacterial genera, such as *Enterococcus*, *Fusicatenibacter*, *Faecalibacterium*, *Roseburia*, *Lachnoclostridium, Tyzzerella_3*, *Butyricicoccus*, and *Dorea*, were enriched at different stages of gastric carcinogenesis.

Wang et al. (33) observed increased bacterial overgrowth and diversification of the microbial communities in gastric cancer patients, with enrichment of *Lactobacillus*, *Escherichia-Shigella*, *Nitrospirae*, *Burkholderia fungorum*, and *Lachnospiraceae* uncultured genera. Park et al. (26, 27) identified two microbial modules (pink and brown) positively correlated with intestinal metaplasia (a precancerous lesion), containing bacteria like *Neisseriaceae*, *Pasteurellaceae*, *Veillonellaceae*, *Pseudomonadaceae*, and *Staphylococcaceae.*

Watanabe et al. (28) found that *Helicobacter pylori* (*H. pylori*) infection was associated with reduced richness and evenness of gastric bacteria, and eradication of *H. pylori* only partially restored microbial diversity. Genera like *Blautia, Ralstonia, Faecalibacterium, Methylobacterium, and Megamonas* were depleted in *H. pylori*-positive patients.

### Gut microbiome and treatment response

Multiple research studies have investigated the correlation between gut microbiota and the effectiveness of cancer therapies. Han et al. (23) found that the gut microbiota composition influenced the effectiveness of several therapies (chemotherapy, immunotherapy, and combination therapy) in HER2-negative advanced gastric cancer patients. Increased levels of *Lactobacillus* were linked to improved outcomes with anti-PD-1/PD-L1 immunotherapy and extended progression-free survival (PFS). Yuan et al. (30) discovered that antibiotic-induced changes in gut bacteria lowered the effectiveness of 5-Fluorouracil (5-FU) treatment for colorectal cancer. Probiotics did not notably improve the treatment’s efficiency compared to 5-FU alone. Zheng et al. (29) found that a probiotic mix with *Bifidobacterium infantis*, *Lactobacillus acidophilus, Enterococcus faecalis, and Bacillus cereus* decreased inflammation, boosted immunity, and improved gut microbial balance in gastric cancer patients following partial gastrectomy.

### Gut microbiome and cancer prevention

Jones et al. (24) demonstrated that the *H. pylori* virulence factor CagA disrupted the gut microbiota balance and promoted the proliferation of epithelial cells in a Drosophila gut model. Addressing the microbial imbalance may minimize the risk of stomach cancer during *H. pylori* infection. Oh et al. (38) discovered that administering probiotics with *H. pylori* eradication treatment reduced the disturbance of gut microbiota induced by antibiotics, potentially lowering the risk factors linked to the onset of gastric cancer. Song et al. (39) demonstrated that pretreatment with the probiotic mixture Bifico improved colitis and decreased tumor development in a mouse model of colitis-associated colorectal cancer (CAC) by changing the gut microbiota composition and lowering the expression of pro-inflammatory genes. In a case–control study, Turati et al. (35) found that consuming a high amount of *Galactooligosaccharide raffinose* was linked to a decreased likelihood of developing stomach cancer.

### Biomarkers and prediction models

Qi et al. (37) developed a random forest model using differential gut bacteria that could predict poorly differentiated colorectal cancer with 100% accuracy, with *Pseudoramibacter, Megamonas,* and *Bifidobacterium* being the most critical bacterial predictors.

Yu et al. (31) described the development of gastric acid-responsive ROS nanogenerators that effectively eliminated *H. pylori* infection in mice without disrupting the normal gut microbiota, suggesting a potential approach for treating *H. pylori* infection and preventing gastric cancer development.

In summary, this systematic review highlights the crucial role of the gut microbiome in gastric and gastrointestinal cancers. Dysbiosis and altered microbial composition were associated with gastric cancer development, precancerous lesions, and treatment response. Specific bacterial genera, such as *Lactobacillus, Bifidobacterium*, and *Akkermansia,* were linked to favorable outcomes, while others, like *Streptococcus* and Escherichia-Shigella, were associated with poorer prognosis. Probiotics and targeted interventions to modulate the gut microbiome showed promising cancer prevention results and enhanced treatment efficacy. Additionally, gut microbial signatures could serve as potential biomarkers for cancer diagnosis, patient stratification, and prediction of treatment response.

## Discussion

Gastric cancer is ranked as the fifth cancer in incidence and fourth in cancer mortality cause. The last report from 2020 shows the incidence of over 1 million new cases per year and more than 768/000 deaths annually. It is estimated that the statistics will rise with all efforts, and in recent years, the incidence of young adults has been increasing (2). Despite the progression of therapeutic methods, targeted immunotherapy, and combinations of chemotherapy or radiotherapy with surgery, the challenge with unresectable advanced-stage or progressive metastatic tumors remains a primary concern. Beyond all of these efforts, because of the complexity of the disease and heterogeneity of tumors, the efforts to overcome the therapy failures and find the underlying causes are still ongoing. Among the factors known so far, some studies have investigated the relationship between gut microbiome and the development, course, treatment, and prognosis of gastric cancer.

The effect of gut microbiota on immune system response is already known (40). Beyond this critical role in the immune system hemostasis, alteration in the normal composition of microbiomes taxa proved to be a predisposing factor in many diseases such as colorectal cancer, Cardiovascular disease, and inflammatory bowel disease (41). This alteration is called dysbiosis. The process may promote hyperplasia and inflammation of the human tissues and lead to neoplasia. *H. pylori*, as a known etiological factor for gastric cancer and precancerous lesions, *H. pylori* can produce dysbiosis in the gastric and intestinal mucosa (42). However, the extent of this change and its probable effect on the development of neoplasia has not been studied well. Jones et al. (24) studied the effect of the cagA protein factor on the midgut microbiome of the germ-free adult Drosophila model. Cytotoxin-associated gene A (cagA) is a virulence protein that, by injection in the cytoplasm of gastric cells arising from *H. pylori* infection, may start the oncogenic potency of this bacteria. Drosophila cagA transgenic models and the protein expression were found to be enough for starting the excessive proliferation process and may contribute to dysbiosis of gut microbiota. Also, this phenomenon elicits the expression of innate immune components, Diptericin and Duox.

The effect of *H. pylori* on dysbiosis was studied on human patients with gastric cancer or precancerous lesions, too. Wang et al. (34) proved the alteration of human gut microbiota by investigating the 313 feces of patients by metagenomics sequencing. The infected patients with *H. Pylori* showed the colonization of *P. corpi* and that Vitamine B 12 levels decreased among the group. *P. corpi* was known before as a microorganism associated with rheumatoid arthritis onset and severity (43). The authors did not investigate the inflammation or histological changes in gastric mucosa, and the patients were checked by breathing test. To improve the data showing the relationship between dysbiosis and GC formation, Turati et al. (35) they investigated the effect of prebiotics on the risk of gastric and upper digestive tract cancers. Prebiotics are substrates that are selectively utilized by microbiota to maintain the health of the digestive system. In a case–control study of nearly 17 years, among several prebiotics used by participants, only high raffinose (a kind of galactooligosaccharide) intake reduced the risk of GC formation. The authors declared that because of the low data, the relationship between the two factors is not strong and needs more investigation. These data showed that there should be a relationship between microbiota in the digestive system environment and the carcinogenesis process. In addition, Song et al. (36) test the idea of using probiotics to decrease inflammation in mice models of IBD.

Bifico, an approved over-the-counter drug in China, previously showed relief from gastritis induced by *H. pylori* in mice and experimental colitis in mice (44, 45). The article examines the impact of this probiotic on malignancies linked with colitis. The results showed that Bifico reduced intestinal inflammation and inhibited tumor formation. The 16 s rRNA sequencing revealed that the supplementation reduced the prevalence of *Desulfovibrio, Mucispirillum, Odoribacter*, and Lactobacillus. In addition to that impact, the Bifico target taxa may engage with CXCL1, CXCL2, CXCL3, and CXCL5, all ligands of CXC motif receptor 2 (CXCR2). All these effects reduced the development of colorectal cancer in mice with an inflammatory bowel disease model. If the idea is investigated in the field of GC, the findings might be positive, even if such research has not been published yet (46).

Moving further, Wang et al. (33) Indicated that changes in gut flora occur in gastric cancer. Changes involve diversifying microorganism populations, increasing bacterium number, and enriching bacteria with possible cancer-promoting actions. *Proteobacteria, Bacillota, Bacteroidota, Fusobacteria,* and *Actinobacteria* were the predominant bacterial phyla in the microbiome of cancer patients, as revealed by compositional analysis. Supporting the idea, Park et al. (26) investigated the hypothesis with the same method on patients with gastritis, intestinal metaplasia, and gastric cancer. The abundance of *Rhizobiales* increased as *H. pylori*-infected patients advanced from gastritis to intestinal metaplasia, alongside changes in microbiota across all patient groups. T4SS genes were prominently detected in the metagenome of patients in the metaplasia stage using RNA sequencing and analysis. The T4SS gene encodes proteins that facilitate the injection of cagA protein into the cytoplasm of infected gastric epithelium. The results indicate that elevated levels of these variables might stimulate the development of cancer. The study’s findings on the alterations in microbiota following the eradication of *H. pylori* are significant. While the microbiome makeup of individuals with successfully eradicated *H. pylori* and those without *H. pylori* showed similarities, but there was no difference in the relative abundance of T4SS genes between the two groups. This gene is crucial in establishing *H. pylori* colonization and the progression of severe outcomes in individuals infected with virulent strains. Patients with gastritis or metaplasia were examined to analyze the correlation between *H. pylori* and other T4SS gene-contributing bacteria such as *Rhizobiales* and *Neisseriaceae*. The relative quantity of these two species grew as the abundance of *H. pylori* dropped. The latest study’s results corroborate Jones et al.’s research on the drosophila model. Miao et al. (21) similarly found the changes during the stages of formation of gastric neoplasms.

In contrast with the recently mentioned article, they analyze the feces of patients with superficial gastritis (SG), atrophic gastritis (AG), gastric mucosal atypical hyperplasia (GMAH), and advanced gastric cancer (GC). Through 16 s rRNA gene sequencing, researchers identified six species and two metabolic pathways that were more prevalent in the cancer group compared to the non-cancer group. The six genera with a high GC content are *Porphyromonas, Scardovia, Halomonas, Actinobacteria_unclassified, Bergeyella*, and *Enterococcus*.

GC has not yet studied the relationship between gut microbiota-specific organisms and the type of neoplasms. Despite this fact, the study of Qi et al. (37) on colorectal cancer (CRC) patients showed the differences between flora in poorly and well-differentiated CRC. The fecal samples from patients were tested and compared using sequencing. *Blautia, Escherichia-Shigella, Streptococcus, Lactobacillus,* and *Bacteroides bacteria* were prevalent in several colorectal cancer individuals with low to moderate differentiation. Nine bacteria were found to have a high abundance in the weakly differentiated group, including *Bifidobacterium, norank_f__Oscillospiraceae,* and *Eisenbergiella*. This term might be used for upcoming research on the subject of GC.

It is essential to consider specific characteristics while examining the research on the relationship between gut microbiota and GC. The T4SS gene’s presence increased in organisms as the sickness progressed, regardless of the variety of bacteria examined. Future research should comprehensively investigate the pathways and mechanisms of the cagA protein and T4SS gene. Park et al. (27) used weighted correlation network analysis to identify two modules linked to advanced gastric carcinogenesis (group C [intestinal metaplasia with *H. pylori* infection] or group D [intestinal metaplasia without *H. pylori* infection]): the pink and brown modules. The two modules included nitrosating/nitrate-reducing bacteria and T4SS protein gene-contributing bacteria from different families such as *Acidobacteriaceae, Burkholderiaceae, Neisseriaceae, Pasteurellaceae, Veillonellaceae, Bartonellaceae, Brucellaceae, unclassified Rhizobiales, Pseudomonadaceae, Sphingomonadaceae, Staphylococcaceae*, and *Xanthomonadaceae*. Group C patients saw reduced *H. pylori* levels and increased intragastric acidity compared to group B, which had *H. pylori* infection without intestinal metaplasia. Bacteria other than *H. pylori* from the pink or brown modules can be identified in group C patients. In group D, the presence of *H. pylori* decreases, but non-*H. pylori* bacteria may increase. Bacteria, not *H. pylori* from the pink or brown modules, are more easily identified in groups C and D than in groups A or B. Both groupings may be associated with the pink and brown modules. Individuals harboring pink- or blue-module bacteria are at a higher risk of developing stomach cancer compared to those in groups A and B. They suggested that using these components might streamline patient screening for endoscopy. Researchers recommend doing more research to determine the specific bacterial taxa associated with the development of stomach cancer due to their presence in the samples.

We believe it is crucial to note that neither research had a substantial sample size. Furthermore, the investigations do not account for distinctions in ethnicity and race. Differences in *H. pylori* prevalence among nations may influence the outcomes of similar investigations. Furthermore, in addition to identifying *H. pylori* as a carcinogenic predisposing factor, future research may uncover other bacteria responsible for this process.

The process of dysbiosis may occur after the eradication of *H. pylori* via antibiotics. Watanabe et al. (32) showed that dysbiosis may persist long-term after eradicating *H. pylori*. In a trial, they compared patients in the early stages of GC who undergo endoscopic submucosal dissection. The changes beyond the *H. pylori* positive group stand longer than negative ones, and they declare that this can involve the development of primary and metachronous GCs. Several genera, including *Blautia, Ralstonia, Faecalibacterium, Methylobacterium*, and *Megamonas,* are deleted among *H. pylori*-positive patients and not restored fully after the eradication and application of the antibiotic regimens. Yu et al. (31) designed an experimental study to prevent this event and decrease dysbiosis during *H. pylori* eradication. Bacteria are vulnerable to oxidative damage caused by external reactive oxygen species (ROS). By creating a ROS nanogenerator, they are testing this theory. The nanogenerators demonstrated the ability to eradicate multi-drug resistant *H. pylori* in mice with little adverse effects. The alterations in the natural flora due to antibiotic therapy were minimal.

Zheng et al. (29) studied patients who underwent partial gastrectomy for the treatment of gastric cancers. Patients who received probiotics supplementation in a randomized controlled experiment had a substantial improvement in immune responses and a decrease in inflammation. The probiotic mixture notably increased the populations of beneficial bacteria such as *Bacteroides, Faecalibacterium*, and *Akkermansia* while reducing the abundance of *Streptococcus*, a pathogenic bacterium associated with cancer development. The study utilized a probiotic blend of *Bifidobacterium infantis, Lactobacillus acidophilus, Enterococcus faecalis,* and *Bacillus cereus*. This combination reduced physiological issues resulting from gastrectomy by monitoring blood levels, index, and microbiological diversity through high-throughput sequencing.

We are in the early stages of using gut microbiota to treat resistant gastric cancers. Chemotherapies have been the most effective therapy for advanced gastric or gastroesophageal junction (GEJ) adenocarcinoma for many years. The therapy’s outcomes are restricted. Immune checkpoint inhibitors (ICI) have revolutionized the treatment of gastric cancer. The limited use of this technology remains a difficulty (47). As far as we know, In melanoma, the gut microbiota can influence the response to anti-PD-1/PD-L1 immunotherapy by infiltrating CD8+ T-cells (48). Based on this theory and to find new methods to overcome the resistance in therapy, Han et al. (23) investigated the relationship between gut microbiota and the response to treatment in HER2-negative advanced gastric cancer.

Patients in stages III or IV according to TNM staging were included in a cohort study. Patients were categorized into three groups based on their treatment method: chemotherapy alone, immunotherapy, and a combination of both. The result data is categorized into responders and non-responders. The study revealed that the gut microbiota can impact the response to therapy in HER-2 negative advanced gastric cancer based on the treatment method. The combo treatment yielded better results. Patients with a greater prevalence of *Lactobacillus* had increased microbial diversity and showed enhanced responsiveness to anti-PD-1/PD-L1 therapy.

Moreover, they frequently encounter enhanced progression-free survival. This might serve as a focal point for developing novel therapeutic approaches to enhance patient outcomes and longevity in the future. Like this study, Yuan et al. (30) revealed the influence of dysbiosis on the anti-tumor efficacy of 5-Fluorouracil (5-FU) treatment in mice models of colorectal cancer. The results suggested that using antibiotics not only proceeds to dysbiosis but can also decrease the treatment efficacy of 5-FU. As we mentioned before, the changes in gut microbiota in this study, after using antibiotics, to the pathogenic microorganisms like *backichia, shigella,* and *Enterobacter* mentioned. The mice that received probiotics with antibiotics showed better anti-tumor activity of 5-FU.

Immunotherapeutic targets for GC have been identified, leading to the development of drugs that specifically target Human Epidermal Growth Factor Receptor 2 (HER2), Vascular Endothelial Growth Factor Receptor 2 (VEGFR2), cytotoxic T-lymphocyte-associated antigen 4 (CTLA-4), PD-1, and programmed cell death ligand 1 (PD-L1). These drugs are currently either available in the market or undergoing clinical trials. Microorganisms have the ability to impact the way medicines are metabolized by undergoing chemical alterations (48) and accumulating them in their bodies. Several studies have verified that the microbiota and their metabolites can significantly influence anti-GC immunotherapy through the release of cytokines and the promotion of T cell infiltration (49). Thus, the use of antibiotics can hinder the effectiveness of cancer immunotherapy due to their impact on the gut microbiota, which plays a crucial role in enhancing the body’s immune response against tumors. Scientists utilized fecal sample sequencing techniques to distinguish between individuals who responded to ICIs and those who did not. They demonstrated that certain microorganisms could potentially be associated with enhanced immunity and the infiltration of immune cells in malignancies (50). For instance, research has shown that gut microorganisms are more abundant in patients who have a robust response to anti-PD-1 therapy. These microbes also stimulate the production of memory CD8+ T cells and natural killer cells in advanced non-small cell lung cancer (NSCLC) patients in China (51). The gut microbiota has the ability to affect the response rate to immunotherapy through several methods, therefore playing a role in the gut microbiota-immune system axis. Gastric cancer (GC) can be categorized into four distinct types: EBV-positive, microsatellite instability (MSI), genomically stable, and chromosomal instability (52). An extensive analysis of microbial profiles in gastric cancer (GC) from two different cohorts has revealed that Selenomonas, Bacteroids, and Porphyromonas are the three most prevalent microorganisms in patients with high microsatellite instability (MSI-high) GC. A clinical experiment aimed to establish a connection between molecular characterisation and the effectiveness of immunotherapy using pembrolizumab, a PD-1 inhibitor. The trial showed that having a high degree of microsatellite instability or being positive for EBV can be used as a predictive marker. Moreover, besides the presence of high-level microsatellite instability and positive EBV status, Hp infection serves as both an indicator of elevated PD-L1 expression and a predictor of unfavorable outcomes following immunotherapy. This is due to its ability to hinder innate and adaptive immune responses, suggesting that Hp infection could be utilized as a measure to assess the effectiveness of immunotherapy in patients with GC. Nevertheless, the specific mechanisms by which Hp regulates immunotherapy are still unclear due to the conflicting findings reported in the aforementioned research (47).

This systematic review conducted a comprehensive analysis of the alterations in the gut microbiota that are associated with the onset of gastric cancer. The text emphasized the complex connection between microbial diversity and cancer, the potential of medicines focusing on the microbiome, and the importance of rigorous methodology in future study. The constraints of this study, such as insufficient data, potential bias, and the inability to incorporate all pertinent components, underscore the necessity for extensive-scale studies. These limitations emphasize the necessity of doing extensive research to validate these findings and delve deeper into the involvement of the microbiome in gastric cancer. Future research should prioritize doing long-term cohort studies to investigate the dynamic alterations in the gut microbiota throughout the development and advancement of gastric cancer. Simultaneously, it is imperative to perform investigations on the pathogenic mechanisms to gain insight into how certain bacteria contribute to or impact the progression of gastric cancer. Furthermore, interventional studies could be carried out to assess the effectiveness of particular microorganisms in preventing and treating stomach cancer. By doing this research, we can get a more comprehensive comprehension of the microbiome’s function in stomach cancer and offer direction for focused preventive and therapy methods in the clinical context.

To conclude, the effect of dysbiosis of gut microbiota on carcinogenesis of GC and the effect of *H. pylori* on gut microbiota should considered. However, as we mentioned, the knowledge is incomplete, and the exact mechanisms have not been studied well. However, knowing the microorganisms that perhaps co-related with *H. Pylori* in GC formation can make them a suitable target for therapy in the future. There is a long way to go.

## Data availability statement

The original contributions presented in the study are included in the article/supplementary material, further inquiries can be directed to the corresponding author.

## Author contributions

AM: Writing – original draft, Writing – review & editing. SH: Writing – original draft, Writing – review & editing. SM: Writing – original draft, Writing – review & editing. RK: Writing – original draft, Writing – review & editing. SMA: Writing – original draft, Writing – review & editing. YD: Writing – original draft, Writing – review & editing. PL: Writing – original draft, Writing – review & editing. SV: Writing – original draft, Writing – review & editing. FJ: Writing – original draft, Writing – review & editing. ShA: Writing – original draft, Writing – review & editing. KN: Writing – original draft, Writing – review & editing. SS: Writing – original draft, Writing – review & editing. NH: Writing – original draft, Writing – review & editing. ArA: Conceptualization, Methodology, Investigation, Writing – original draft, Writing – review & editing. AiA: Methodology, Investigation, Writing – original draft, Writing – review & editing. SS: Conceptualization, Investigation, Supervision, Writing – original draft, Writing – review & editing. MA: Conceptualization, Investigation, Supervision, Writing – original draft, Writing – review & editing.

## Footnotes

^1^ https://jbi.global/

## References

[1] Ferlay J Colombet M Soerjomataram I Parkin DM Piñeros M Znaor A . Cancer statistics for the year 2020: An overview. Int J Cancer. (2021) 149:778–89. doi: 10.1002/ijc.33588 33818764

[2] Lu L Mullins CS Schafmayer C Zeissig S Linnebacher M. A global assessment of recent trends in gastrointestinal cancer and lifestyle-associated risk factors. Cancer Commun (Lond). (2021) 41:1137–51. doi: 10.1002/cac2.12220, PMID: 34563100 PMC8626600

[3] Tan P Yeoh KG. Genetics and molecular pathogenesis of gastric adenocarcinoma. Gastroenterology. (2015) 149:1153–62.e3. doi: 10.1053/j.gastro.2015.05.059 26073375

[4] Correa P. Gastric cancer: overview. Gastroenterol Clin N Am. (2013) 42:211–7. doi: 10.1016/j.gtc.2013.01.002, PMID: 23639637 PMC3995345

[5] Conti CB Agnesi S Scaravaglio M Masseria P Dinelli ME Oldani M . Early gastric Cancer: update on prevention, diagnosis and treatment. Int J Environ Res Public Health. (2023) 20:2149. doi: 10.3390/ijerph20032149, PMID: 36767516 PMC9916026

[6] Lauren P. The two histological main types of gastric carcinoma: DIFFUSE and so-called intestinal-type carcinoma. An attempt at a HISTO-clinical classification. Acta Pathol Microbiol Scand. (1965) 64:31–49. doi: 10.1111/apm.1965.64.1.31 14320675

[7] Nagtegaal ID Odze RD Klimstra D Paradis V Rugge M Schirmacher P . The 2019 WHO classification of tumours of the digestive system. Histopathology. (2020) 76:182–8. doi: 10.1111/his.13975, PMID: 31433515 PMC7003895

[8] van den Ende T Ter Veer E Machiels M Mali RMA Abe Nijenhuis FA de Waal L . The efficacy and safety of (neo)adjuvant therapy for gastric Cancer: a network Meta-analysis. Cancers (Basel). (2019) 11:80. doi: 10.3390/cancers11010080 30641964 PMC6356558

[9] Fuchs CS Doi T Jang RW Muro K Satoh T Machado M . Safety and efficacy of Pembrolizumab monotherapy in patients with previously treated advanced gastric and gastroesophageal junction Cancer: phase 2 clinical KEYNOTE-059 trial. JAMA Oncol. (2018) 4:e180013. doi: 10.1001/jamaoncol.2018.0013, PMID: 29543932 PMC5885175

[10] Kang YK Boku N Satoh T Ryu MH Chao Y Kato K . Nivolumab in patients with advanced gastric or gastro-oesophageal junction cancer refractory to, or intolerant of, at least two previous chemotherapy regimens (ONO-4538-12, ATTRACTION-2): a randomised, double-blind, placebo-controlled, phase 3 trial. Lancet. (2017) 390:2461–71. doi: 10.1016/S0140-6736(17)31827-5 28993052

[11] Shitara K Yatabe Y Matsuo K Sugano M Kondo C Takahari D . Prognosis of patients with advanced gastric cancer by HER2 status and trastuzumab treatment. Gastric Cancer. (2013) 16:261–7. doi: 10.1007/s10120-012-0179-9 22797858

[12] De Vita F Borg C Farina G Geva R Carton I Cuku H . Ramucirumab and paclitaxel in patients with gastric cancer and prior trastuzumab: subgroup analysis from RAINBOW study. Future Oncol. (2019) 15:2723–31. doi: 10.2217/fon-2019-0243 31234645

[13] Zhang XY Zhang PY. Gastric cancer: somatic genetics as a guide to therapy. J Med Genet. (2017) 54:305–12. doi: 10.1136/jmedgenet-2016-104171 27609016

[14] Yu T Guo F Yu Y Sun T Ma D Han J . *Fusobacterium nucleatum* promotes Chemoresistance to colorectal Cancer by modulating autophagy. Cell. (2017) 170:548–63.e16. doi: 10.1016/j.cell.2017.07.008, PMID: 28753429 PMC5767127

[15] Yi Y Shen L Shi W Xia F Zhang H Wang Y . Gut microbiome components predict response to Neoadjuvant Chemoradiotherapy in patients with locally advanced rectal Cancer: a prospective. Longitudinal Study Clin Cancer Res. (2021) 27:1329–40. doi: 10.1158/1078-0432.CCR-20-3445, PMID: 33298472

[16] Thursby E Juge N. Introduction to the human gut microbiota. Biochem J. (2017) 474:1823–36. doi: 10.1042/BCJ20160510, PMID: 28512250 PMC5433529

[17] Png CW Lee WJJ Chua SJ Zhu F Yeoh KG Zhang Y. Mucosal microbiome associates with progression to gastric cancer. Theranostics. (2022) 12:48–58. doi: 10.7150/thno.65302, PMID: 34987633 PMC8690935

[18] Nicholson JK Holmes E Kinross J Burcelin R Gibson G Jia W . Host-gut microbiota metabolic interactions. Science. (2012) 336:1262–7. doi: 10.1126/science.1223813 22674330

[19] Lertpiriyapong K Whary MT Muthupalani S Lofgren JL Gamazon ER Feng Y . Gastric colonisation with a restricted commensal microbiota replicates the promotion of neoplastic lesions by diverse intestinal microbiota in the *Helicobacter pylori* INS-GAS mouse model of gastric carcinogenesis. Gut. (2014) 63:54–63. doi: 10.1136/gutjnl-2013-305178, PMID: 23812323 PMC4023484

[20] Coker OO Dai Z Nie Y Zhao G Cao L Nakatsu G . Mucosal microbiome dysbiosis in gastric carcinogenesis. Gut. (2018) 67:1024–32. doi: 10.1136/gutjnl-2017-314281, PMID: 28765474 PMC5969346

[21] Miao Y Tang H Zhai Q Liu L Xia L Wu W . Gut microbiota Dysbiosis in the development and progression of gastric Cancer. J Oncol. (2022) 2022:1–15. doi: 10.1155/2022/9971619 PMC9441395 36072968

[22] Page MJ McKenzie JE Bossuyt PM Boutron I Hoffmann TC Mulrow CD . The PRISMA 2020 statement: an updated guideline for reporting systematic reviews. Rev Esp Cardiol (Engl Ed). (2021) 74:790–9. doi: 10.1016/j.recesp.2021.06.016, PMID: 34446261

[23] Han Z Cheng S Dai D Kou Y Zhang X Li F . The gut microbiome affects response of treatments in HER2-negative advanced gastric cancer. Clin Transl Med. (2023) 13:e1312. doi: 10.1002/ctm2.1312, PMID: 37381590 PMC10307992

[24] Jones TA Hernandez DZ Wong ZC Wandler AM Guillemin K. The bacterial virulence factor CagA induces microbial dysbiosis that contributes to excessive epithelial cell proliferation in the Drosophila gut. PLoS Pathog. (2017) 13:e1006631. doi: 10.1371/journal.ppat.1006631, PMID: 29049360 PMC5648253

[25] Oh B Kim BS Kim JW Kim JS Koh SJ Kim BG . The effect of probiotics on gut microbiota during the *Helicobacter pylori* eradication: randomized controlled trial. Helicobacter. (2016) 21:165–74. doi: 10.1111/hel.12270 26395781

[26] Park CH Lee AR Lee YR Eun CS Lee SK Han DS. Evaluation of gastric microbiome and metagenomic function in patients with intestinal metaplasia using 16S rRNA gene sequencing. Helicobacter. (2019) 24:e12547. doi: 10.1111/hel.12547, PMID: 30440093 PMC6587566

[27] Park CH Lee JG Lee AR Eun CS Han DS. Network construction of gastric microbiome and organization of microbial modules associated with gastric carcinogenesis. Sci Rep. (2019) 9:12444. doi: 10.1038/s41598-019-48925-4, PMID: 31455798 PMC6712011

[28] Watanabe M Otake R Kozuki R Toihata T Takahashi K Okamura A . Recent progress in multidisciplinary treatment for patients with esophageal cancer. Surg Today. (2020) 50:12–20. doi: 10.1007/s00595-019-01952-0, PMID: 31535225 PMC6952324

[29] Zheng C Chen T Wang Y Gao Y Kong Y Liu Z . A randomised trial of probiotics to reduce severity of physiological and microbial disorders induced by partial gastrectomy for patients with gastric cancer. J Cancer. (2019) 10:568–76. doi: 10.7150/jca.29072, PMID: 30719153 PMC6360416

[30] Yuan L Zhang S Li H Yang F Mushtaq N Ullah S . The influence of gut microbiota dysbiosis to the efficacy of 5-fluorouracil treatment on colorectal cancer. Biomed Pharmacother. (2018) 108:184–93. doi: 10.1016/j.biopha.2018.08.165, PMID: 30219675

[31] Yu J Guo Z Yan J Bu C Peng C Li C . Gastric acid-responsive ROS Nanogenerators for effective treatment of *Helicobacter pylori* infection without disrupting homeostasis of intestinal Flora. Adv Sci (Weinh). (2023) 10:e2206957. doi: 10.1002/advs.202206957, PMID: 37127895 PMC10369278

[32] Watanabe T Nadatani Y Suda W Higashimori A Otani K Fukunaga S . Long-term persistence of gastric dysbiosis after eradication of *Helicobacter pylori* in patients who underwent endoscopic submucosal dissection for early gastric cancer. Gastric Cancer. (2021) 24:710–20. doi: 10.1007/s10120-020-01141-w, PMID: 33201352 PMC8065006

[33] Wang L Zhou J Xin Y Geng C Tian Z Yu X . Bacterial overgrowth and diversification of microbiota in gastric cancer. Eur J Gastroenterol Hepatol. (2016) 28:261–6. doi: 10.1097/MEG.0000000000000542, PMID: 26657453 PMC4739309

[34] Wang D Li Y Zhong H Ding Q Lin Y Tang S . Alterations in the human gut microbiome associated with *Helicobacter pylori* infection. FEBS Open Bio. (2019) 9:1552–60. doi: 10.1002/2211-5463.12694, PMID: 31250988 PMC6724102

[35] Turati F Concina F Bertuccio P Fiori F Parpinel M Garavello W . Prebiotics and the risk of upper digestive tract and stomach cancers: the PrebiotiCa study. J Acad Nutr Diet. (2023) 123:1772–80. doi: 10.1016/j.jand.2023.07.008, PMID: 37468063

[36] Song H Wang W Shen B Jia H Hou Z Chen P . Pretreatment with probiotic Bifico ameliorates colitis-associated cancer in mice: transcriptome and gut flora profiling. Cancer Sci. (2018) 109:666–77. doi: 10.1111/cas.13497, PMID: 29288512 PMC5834773

[37] Qi Z Zhibo Z Jing Z Zhanbo Q Shugao H Weili J . Prediction model of poorly differentiated colorectal cancer (CRC) based on gut bacteria. BMC Microbiol. (2015) 21:312. doi: 10.1186/s12866-022-02712-w, PMID: 36539710 PMC9764708

[38] Oh HY Kim B-S Seo S-S Kong J-S Lee S-Y Park K-M . The association of uterine cervical microbiota with an increased risk for cervical intraepithelial neoplasia in Korea. Clinical Microbiology and Infection. (2015) 21:674-e1. 10.1016/j.cmi.2015.02.026 25752224

[39] Song D Peng Q Chen Y Zhou X Zhang F Li A . Altered gut microbiota profiles in sows and neonatal piglets associated with porcine epidemic diarrhea virus infection. Scientific reports. (2017) 7:17439., PMID: 29234140 10.1038/s41598-017-17830-z PMC5727058

[40] Wang J Zhu N Su X Gao Y Yang R. Gut-microbiota-derived metabolites maintain gut and systemic immune homeostasis. Cells. (2023) 12:793. doi: 10.3390/cells12050793 36899929 PMC10000530

[41] Holmes E Li JV Marchesi JR Nicholson JK. Gut microbiota composition and activity in relation to host metabolic phenotype and disease risk. Cell Metab. (2012) 16:559–64. doi: 10.1016/j.cmet.2012.10.007, PMID: 23140640

[42] Wroblewski LE Peek RM Jr. *Helicobacter pylori*, Cancer, and the gastric microbiota. Adv Exp Med Biol. (2016) 908:393–408. doi: 10.1007/978-3-319-41388-4_19 27573782

[43] Zhang X Zhang D Jia H Feng Q Wang D Liang D . The oral and gut microbiomes are perturbed in rheumatoid arthritis and partly normalized after treatment. Nat Med. (2015) 21:895–905. doi: 10.1038/nm.3914, PMID: 26214836

[44] Yu HJ Liu W Chang Z Shen H He LJ Wang SS . Probiotic BIFICO cocktail ameliorates *Helicobacter pylori* induced gastritis. World J Gastroenterol. (2015) 21:6561–71. doi: 10.3748/wjg.v21.i21.6561, PMID: 26074694 PMC4458766

[45] Zhao HM Huang XY Zuo ZQ Pan QH Ao MY Zhou F . Probiotics increase T regulatory cells and reduce severity of experimental colitis in mice. World J Gastroenterol. (2013) 19:742–9. doi: 10.3748/wjg.v19.i5.742, PMID: 23430765 PMC3574601

[46] Komori E Kato-Kogoe N Imai Y Sakaguchi S Taniguchi K Omori M . Changes in salivary microbiota due to gastric cancer resection and its relation to gastric fluid microbiota. Sci Rep. (2023) 13:15863. doi: 10.1038/s41598-023-43108-8 37740058 PMC10516953

[47] Kim ST Cristescu R Bass AJ Kim KM Odegaard JI Kim K . Comprehensive molecular characterization of clinical responses to PD-1 inhibition in metastatic gastric cancer. Nat Med. (2018) 24:1449–58. doi: 10.1038/s41591-018-0101-z, PMID: 30013197

[48] Gopalakrishnan V Spencer CN Nezi L Reuben A Andrews MC Karpinets TV . Gut microbiome modulates response to anti-PD-1 immunotherapy in melanoma patients. Science. (2018) 359:97–103. doi: 10.1126/science.aan4236, PMID: 29097493 PMC5827966

[49] Zimmermann M Zimmermann-Kogadeeva M Wegmann R Goodman AL. Mapping human microbiome drug metabolism by gut bacteria and their genes. Nature. (2019) 570:462–7. doi: 10.1038/s41586-019-1291-3, PMID: 31158845 PMC6597290

[50] Jin Y Dong H Xia L Yang Y Zhu Y Shen Y . The diversity of gut microbiome is associated with favorable responses to anti-programmed death 1 immunotherapy in Chinese patients with NSCLC. J Thorac Oncol. (2019) 14:1378–89. doi: 10.1016/j.jtho.2019.04.007 31026576

[51] Oster P Vaillant L Riva E McMillan B Begka C Truntzer C . *Helicobacter pylori* infection has a detrimental impact on the efficacy of cancer immunotherapies. Gut. (2022) 71:457–66. doi: 10.1136/gutjnl-2020-323392, PMID: 34253574 PMC8862014

[52] Comprehensive molecular characterization of gastric adenocarcinoma. Comprehensive molecular characterization of gastric adenocarcinoma. Nature. (2014) 513:202–9. doi: 10.1038/nature13480 10.1038/nature13480 PMC4170219 25079317

